# A meta-analysis of genome-wide association studies using Japanese and Taiwanese has revealed novel loci associated with gout susceptibility

**DOI:** 10.1007/s13577-021-00665-2

**Published:** 2022-01-15

**Authors:** Shun-Jen Chang, Yu Toyoda, Yusuke Kawamura, Takahiro Nakamura, Masahiro Nakatochi, Akiyoshi Nakayama, Wei-Ting Liao, Seiko Shimizu, Tappei Takada, Kenji Takeuchi, Kenji Wakai, Yongyong Shi, Nariyoshi Shinomiya, Chung-Jen Chen, Changgui Li, Yukinori Okada, Kimiyoshi Ichida, Hirotaka Matsuo, for Japan Gout Genomics Consortium (Japan Gout), Yuya Shirai, Kenji Wakai, Toru Shimizu, Hiroshi Ooyama, Keiko Ooyama, Mitsuo Nagase, Yuji Hidaka, Hiroshi Nakashima, Yutaka Sakurai, Masashi Tsunoda

**Affiliations:** 1 Department of Kinesiology, Health and Leisure Studies, National University of Kaohsiung, Kaohsiung, Taiwan; 2 Department of Integrative Physiology and Bio-Nano Medicine, National Defense Medical College, 3-2 Namiki, Tokorozawa, Saitama, 359-8513 Japan; 3 Laboratory for Mathematics, National Defense Medical College, Saitama, Japan; 4 Public Health Informatics Unit, Department of Integrated Health Sciences, Nagoya University Graduate School of Medicine, Aichi, Japan; 5 Department of Biotechnology, College of Life Science, Kaohsiung Medical University, Kaohsiung, Taiwan; 6 Department of Pharmacy, The University of Tokyo Hospital, Tokyo, Japan; 7 Department of Preventive Medicine, Nagoya University Graduate School of Medicine, Aichi, Japan; 8 Bio-X Institutes, Key Laboratory for the Genetics of Developmental and Neuropsychiatric Disorders (Ministry of Education), Shanghai Jiao Tong University, Shanghai, China; 9 Affiliated Hospital of Qingdao University and Biomedical Sciences Institute of Qingdao University (Qingdao Branch of SJTU Bio-X Institutes), Qingdao University, Qingdao, China; 10 Department of Internal Medicine, Kaohsiung Medical University Hospital, Kaohsiung, Taiwan; 11 Department of Endocrinology and Metabolism, The Affiliated Hospital of Qingdao University, Qingdao, China; 12 Department of Statistical Genetics, Osaka University Graduate School of Medicine, Osaka, Japan; 13 Laboratory of Statistical Immunology, Immunology Frontier Research Center (WPI-IFReC), Osaka University, Osaka, Japan; 14 Integrated Frontier Research for Medical Science Division, Institute for Open and Transdisciplinary Research Initiatives, Osaka University, Osaka, Japan; 15 Laboratory for Systems Genetics, RIKEN Center for Integrative Medical Sciences, Yokohama, Japan; 16 Department of Pathophysiology, Tokyo University of Pharmacy and Life Sciences, Tokyo, Japan

To the Editor,

We have recently identified four novel genomic loci influencing gout susceptibility at the genome-wide significance level (*P* < 5.0 × 10^–8^) via genome-wide association study (GWAS) meta-analyses of clinically defined gout with more finely differentiated subtypes in Japanese cohorts [1]. However, there are many loci that are inconclusive but suggestive of an association with the risk of gout. This prompted us to carry out a meta-analysis using previous gout GWASs of the Japanese [1] and the Taiwanese [2] populations. Integration of the results allowed us to focus on 11 SNPs (*P* < 1.0 × 10^–5^ in the Japanese populations in our previous study [1]), for which information for conducting a meta-analysis was available (Supplementary Table S1). As described below, we successfully identified, for we believe the first time, two loci associated with the risk of gout at a genome-wide level of significance.

Details of the study participants, including a total of 3800 gout cases and 6625 controls (Japanese: 3053 cases and 4554 controls; Taiwanese: 747 cases and 2071 controls), were described previously [1, 2]. Regarding the 11 SNPs potentially associated with gout, we obtained association summary statistics data of the SNPs from the published two GWASs and combined them. Our meta-analysis of gout revealed genome-wide significant associations of rs16998073-T (T/A: major allele is A) [intergenic between *PR/SET Domain 8* (*PRDM8*) and *fibroblast growth factor 5* (*FGF5*)] and rs10847689-C (C/T: major allele is T) [intronic in *MLX interacting protein* (*MLXIP*)] with decreased [odds ratio (OR) = 0.835, *P* = 3.02 × 10^−8^] and increased (OR = 1.202, *P* = 3.67 × 10^−8^) the risk of gout, respectively (Table 1).

**Table 1:** Two gout loci that were revealed by a meta-analysis using the Japanese and the Taiwanese populations

SNP*	A1/A2^†^	Chr	Position (bp)^‡^	Gene	Illumina array (Japanese)				Japonica array (Japanese)				Replication study (Taiwanese)				Meta-analysis		
					Case	Ctrl	OR (95% CI)	*P* value	Case	Ctrl	OR (95% CI)	*P* value	Case	Ctrl	OR (95% CI)	*P* value	OR (95% CI)	*P* value	Het*P*^#^
rs16998073	T/A	4	81,184,341	*PRDM8*–*FGF5*	0.273	0.306	0.829 (0.758–0.906)	3.82 × 10^−5^	0.277	0.304	0.859 (0.747–0.988)	3.30 × 10^−2^	0.389	0.435	0.828 (0.734–0.934)	2.16 × 10^−3^	0.835 (0.783–0.890)	3.02 × 10^−8^	0.903
rs10847689	C/T	12	122,613,000	*MLXIP*	0.288	0.243	1.263 (1.155–1.380)	3.03 × 10^−7^	0.276	0.259	1.112 (0.964–1.284)	1.45 × 10^−1^	0.303	0.274	1.153 (1.013–1.313)	3.11 × 10^−2^	1.202 (1.126–1.283)	3.67 × 10^−8^	0.262

*dbSNP rs number

^†^A1, effect allele of which the frequencies are shown in the “Case” and “Ctrl” columns; A2, non-effect allele

^‡^SNP positions are based on NCBI human genome reference sequence Build hg19

^#^When heterogeneity was revealed by statistical testing (Het*P* < 0.05), we implemented the DerSimonian and Laird random-effects model; otherwise, we used the inverse-variance fixed-effects model

Information on all SNPs analyzed in this study is shown in Supplementary Table S1

*SNP* single-nucleotide polymorphism, *Chr* chromosome, *Ctrl* control, *OR* odds ratio, *CI* confidence interval, *HetP* heterogeneity *P* value

Having proceeded on the assumption that the nearest genes to the identified SNPs were likely candidates for causality, our results strongly support the associations of *FGF5* and *MLXIP* with the risk of gout, a urate-related disease. These findings agree with those of recent studies, including our own, which identified *FGF5* as a serum urate-affecting gene [3]. In a previous *trans*-ancestry GWAS of serum urate in 457,690 individuals (including subjects with European ancestry, East Asian ancestry, African Americans, South Asian ancestry, and Hispanics) [4], near loci of the two SNPs we herein focused on (rs10857147 and rs148015593 of which the nearest genes are *FGF5* and *MLXIP*, respectively) were found to be associated with serum urate. The previous study also calculated the gout ORs of their effect-allele {rs10857147, OR = 1.04 [95% confidence interval (CI), 1.01–1.07]; rs148015593, OR = 1.06 (95% CI, 1.04–1.09)}; however, their effects on the risk of gout have hitherto been unclear. We herein provide the first genetic evidence to suggest the pathophysiological importance of *MLXIP* in the context of gout. *MLXIP* encodes glucose-sensitive transcription factor, which is involved in energy metabolism, including the activation of the pentose phosphate pathways [5] that stimulates de novo purine nucleotide synthesis. Genetic variations in *MLXIP* may thus influence the endogenous production of uric acid.

Interestingly, although information on blood pressure was not available in this study, the SNP rs16998073 (upstream of *FGF5*) was reportedly associated with hypertension susceptibility in East Asians [6], in addition to its association with gout as found in this study. Of note, whereas the rs16998073-T allele was associated with a lower risk of gout as shown here, the minor allele (rs16998073-T) is reportedly associated with increased risk of hypertension. These relationships are seemingly paradoxical, given that the elevation of serum urate levels has been thought to be a potential cause of the development of hypertension (although this causality is not conclusive: some Mendelian randomization studies do not support a causal role of serum urate in hypertension [7]). However, such cases can occur, since the influences of a genetic variation on metabolic syndrome components are not always entirely positive or negative. For example, despite a positive association with higher triglyceride levels and the risk of dyslipidemia [8] as well as gout [1], an SNP (rs1260326) in the *glucokinase regulator* (*GCKR*) gene is reportedly protective against type 2 diabetes [8, 9], suggesting the presence of a complex relationship between the components of this metabolic syndrome and their genetic influencers. Hence, via extremely different (independent) molecular bases, genetic variation in *FGF5* may influence the risk of gout and hypertension. There is as yet little molecular evidence to support the role of FGF5—a secretory signaling protein [10]—in the pathogenesis of hyperuricemia/gout as well as hypertension. To address these open questions, further investigations are needed into how the genetic variation in *FGF5* can affect the biological mechanisms related to urate handling or uric acid-mediated inflammatory processes as well as blood pressure.

In conclusion, our results indicate the significant association of *FGF5* and *MLXIP* with gout susceptibility. While further studies are required to clarify this notion, our findings should contribute to a better understanding of the pathophysiology of gout.

## Supplementary Information

Below is the link to the electronic supplementary material.

Supplementary file1 (XLSX 17 KB)
